# Artificial Intelligence cannot Transform Observational Non-difference into Clinical Equivalence

**DOI:** 10.1055/a-2961-0699

**Published:** 2026-09-23

**Authors:** Gabriele Wurm Johansson, Anastasios Koulaouzidis, Ervin Toth

**Affiliations:** 1 Department of GastroenterologySkåne University Hospital, Lund UniversityMalmöSweden; 2 Department of Clinical Research 6174 University of Southern DenmarkSvendborgDenmark; 3 Department of SurgeryOdense University Hospital and Svendborg HospitalSvendborgDenmark; 4 Department of GastroenterologyPomeranian Medical UniversitySzczecinPoland

## 



**To the Editor,**

Nevarez et al. should be commended for applying automated assessment to the unresolved problem of small-bowel (SB) cleanliness. 1 However, their conclusion that a liquid diet provides “adequate” cleanliness comparable to laxatives—and, therefore, questions routine purgative use—is not supported by the design or analysis. The central problem is allocation according to the usual practice of 11 participating hospitals rather than randomisation. The article does not show the centre-by-preparation distribution or account for centre clustering. If centres predominantly used different regimens, preparation may be inseparable from centre, including differences in case mix, instructions, referral pathways, and reading practices. The baseline imbalances in age and diabetes, both subsequently associated with cleanliness, confirm that the groups were not exchangeable. Moreover, *p* = 0.415 does not demonstrate comparability. The study was designed only to detect differences; it did not define in advance how close the results had to be for the “treatments” were genuinely equivalent. Failure to reject a difference is not evidence of equivalence. The problem is compounded because the sample-size calculation concerned a 20% difference in the proportion of “adequate” cleansing, whereas the primary analysis used a continuous Endoclean score. The stated power calculation, therefore, does not justify the analysis performed.

The authors also acknowledge that no validated Endoclean cutoff defines adequate preparation. Nevertheless, “adequate” cleanliness becomes the clinical conclusion. Image-level accuracy of a convolutional neural network does not automatically validate the clinical meaning of a whole-video percentage. 2 No central expert assessment or clinically based threshold was provided. The arbitrary categories further generated discordant results: the four-group categorical comparison was significant, the dichotomised comparison was not, and significance appeared after post hoc pooling of all laxatives against the liquid diet. Moreover, 10 of 19 incomplete examinations occurred with Pleinvue ( *p* = 0.01), a clinically important signal that deserves more than a cleanliness-based dismissal. A recently published study by Ferreira et al. 3 does not validate the broader inference made in this paper. It compared 1 L of water with 1 L of PEG or PEG-ascorbate as post-pyloric boosters after a clear-liquid diet, 12-hour fasting, simethicone, and real-time confirmation that the capsule had entered the SB. It, therefore, addressed booster composition and timing, not liquid diet alone versus laxative preparation. Although overall adequacy and diagnostic yield were not significantly different by omnibus testing, adequate preparation was numerically lower with water (81.9% vs. 87.5% and 91.4%), and third-tertile SB-CLEAR scores were significantly poorer (2.3 vs. 2.5 and 2.6; *p* = 0.021). The protocol specified a 15% non-inferiority margin, but the published results do not present the pairwise effect estimates, and confidence intervals (CIs) needed to establish non-inferiority. Appropriately, the authors describe their findings as hypothesis-generating.

The defensible conclusion is narrower: under nonrandomised, centre-dependent conditions, no statistically significant difference in mean whole-video Endoclean scores was detected among four strategies. These exploratory data do not demonstrate equivalence and should not change practice. A definitive comparison requires concealed randomisation, prespecified and clinically justified non-inferiority margins, pairwise CIs, and validated segmental outcomes linked to lesion visibility and diagnostic performance. 3 4 5 6 Artificial intelligence may standardise measurement; however, it cannot cure confounding or transform statistical non-difference into clinical equivalence.

## Declaration of GenAI Use

During preparation of this letter, the authors used ChatGPT (OpenAI, San Francisco, CA, USA) solely for language editing, stylistic refinement, and document formatting. The tool was not used to generate data, perform analyses, select references, or formulate the scientific arguments or conclusions. All content was critically reviewed, verified, and approved by the authors, who take full responsibility for the accuracy and integrity of the submitted work.
